# Opt-in and opt-out strategies for HIV testing

**DOI:** 10.1016/j.lanepe.2025.101482

**Published:** 2025-10-24

**Authors:** Emilia Miró, Patricia Trenc, Begoña Espinosa, Lourdes Piedrafita, Nayra Cabrera, Concepción Abellas Álvarez

**Affiliations:** a Universitat de Barcelona, Barcelona, Spain; b Emergency Department, Hospital Universitario Miguel Servet, Zaragoza, Spain; c Emergency Department, Hospital de Manacor, Manacor, Spain; d Emergency Department, Hospital General Doctor Balmis de Alicante, Alicante, Spain; e Emergency Department, Hospital Universitario Dr. Negrín, Gran Canaria, Spain; f Pontevedra and O Salnés Health Area, Galician Health Service, National Coordinator of the “Deja tu Huella” Project for Spainsh Emergency Medicine Society (SEMES), Pontevedra, Spain

Miłosz Parczewski and colleagues provide an insightful review of the ongoing challenges in HIV control across Europe.^1^ One crucial aspect that requires further discussion is the choice between opt-in and opt-out HIV testing strategies, particularly in emergency departments. Both approaches have demonstrated benefits and limitations, and their implementation varies widely across countries.

The opt-out strategy, successfully implemented in high-prevalence areas of the UK, has significantly increased HIV diagnosis rates by normalising routine testing and reducing stigma.^2^ Studies have shown that opt-out testing in emergency departments detects undiagnosed cases earlier, improving linkage to care and reducing community transmission. However, this approach faces barriers such as cost, logistics, and ethical concerns, particularly regarding informed consent.^3^

By contrast, the opt-in strategy, widely adopted in Spain through the “Deja tu Huella” programme, ensures voluntary patient participation and aligns with traditional consent models.^4^ This approach has been instrumental in expanding targeted HIV testing in emergency departments, focusing, in addition to the very well-known HIV risk factors, on high-risk populations with specific diagnoses or circumstances where HIV infection is more prevalent. Opt-in testing can have lower uptake rates than opt-out testing, as it depends on physician discretion and patient willingness.^5^

Comparative analyses suggest that opt-out testing leads to higher testing coverage and earlier diagnosis, yet requires substantial resources and political commitment for widespread implementation. Opt-in programmes, while easier to implement, may reinforce disparities if testing is not equitably offered. However, the results obtained in Spain showed that over three years (2021–2023), 130,000 HIV-test were performed in emergency departments, diagnosing 1620 new infections,^4^ whereas in the UK, over two years (2023–2024), 741 new diagnoses were made from 1.9 million tests.^2^ In other words, in Spain, 80 tests were needed to obtain one positive result, whereas in the UK, 2564 tests were required.

A hybrid approach, integrating opt-out testing in high-prevalence settings and enhanced opt-in strategies elsewhere, could balance feasibility and effectiveness. To optimise HIV screening in emergency departments, we recommend expanding opt-out testing where feasible, particularly in high-prevalence areas; strengthening targeted opt-in programmes with clinician training and decision-support tools; and ensuring adequate funding and local, regional, and national policy support to sustain large-scale HIV screening efforts. Adapting HIV testing strategies to local epidemiological and health-care system realities is crucial to achieving the 95–95–95 targets and improving early diagnosis rates across Europe.

## Contributors

EM and CAA were involved in manuscript writing. All authors critically reviewed and approved the final manuscript.

## Declaration of interests

The authors declare no conflicts of interest.

## References

[1] Parczewski M., Gökengin D., Sullivan A. (2025). Control of HIV across the WHO European region: progress and remaining challenges. Lancet Reg Health Eur.

[2] Hill-Tout R. (2024).

[3] Ramiro Avilés M.Á. (2024). Information and consent in HIV screening. Emergencias.

[4] González Del Castillo J., Llorens P., Trenc P. (2024). Red VIH de Urgencias. New recommendations of Spanish Society of Emergency Medicine (SEMES) for emergency department diagnosis of HIV infection based on results from Spain's "Leave Your Mark" program. Emergencias.

[5] Roberts D.A., Bridenbecker D., Haberer J.E., Barnabas R.V., Akullian A. (2022). The impact of prevention-effective PrEP use on HIV incidence: a mathematical modelling study. J Int AIDS Soc.

